# Tooth eruption sequence and dental crowding: a case-control study

**DOI:** 10.12688/f1000research.3196.1

**Published:** 2014-06-06

**Authors:** Vahid Moshkelgosha, Negar Khosravifard, Ali Golkari

**Affiliations:** 1 Orthodontics Research Center and Department of Orthodontics, School of Dentistry, Shiraz University of Medical Sciences, Shiraz, 71956-15878, Iran; 2 School of Dentistry, Department of Oral and Maxillofacial Radiology, Shiraz University of Medical Sciences, Shiraz, 71956-15878, Iran; 3 Department of Dental Public Health, School of Dentistry, Shiraz University of Medical Sciences, Shiraz, 71956-15878, Iran

## Abstract

When cases of dental crowding are identified and diagnosed promptly, interceptive orthodontics is particularly successful.

**Aim:** To assess the differences in the eruption sequence of the mandibular canine and first premolar teeth in children with and without dental crowding.

**Materials and** **Methods:** Children who attended the Shiraz Dental School's orthodontic clinic (Iran) from September to December 2012 were enrolled in this case-control study. Tooth size arch length discrepancy (TSALD) of all 8-10 year olds was calculated from patients’ dental models. Thirty-six children were randomly selected from those with TSALD of equal or less than 4mm (those with crowding). Each selected case was matched for sex and age with another child (as control) with TSALD>−4mm attending the same clinic, in the same time period. The existing panoramic radiographs were traced and the eruption percentages were measured for mandibular canine and first premolar teeth. The mean difference between canine and first premolar eruption percentages was compared between the case and control groups using the SPSS (version PASW 18) software and a paired sample t-test.

**Results:** Canine and first premolar eruption percentages in the case group were 65.82±13.00 and 78.92±10.15 percent, respectively. The mean eruption percentages for canines and first premolars of the control group were 74.12±14.55 and 75.47±11.60 percent, respectively. There was a significant difference in pre-eruptive positions of canine and first premolar teeth in those with moderate to severe crowding when compared to the control group (p<0.001).

**Conclusion:** These findings may improve the early diagnosis of children with high risk of developing moderate to severe crowding during mixed dentition.

## Introduction

The National Health and Nutrition Examination Survey (NHANES III, 1998) reported dental crowding as the most prevalent form of malocclusion among children in the United States, with about 50% having some degrees of crowding in the mixed dentition that worsened as they stepped into adolescence and adulthood ^1^.

Despite the frequent advancements in treatment modalities and the use of high technology equipment in contemporary orthodontics, little attempt has been made to advance preventive orthodontic services. Prevention and interception of orthodontic problems are major concerns as they can improve the quality of life of people and save their money and time ^2, 3^. Preventing a developing malocclusion or intercepting its path is always more economic and less complicated than correcting the resulting malocclusion later ^4, 5^. In many countries, due to the shortage of specialists or the inability of the society to afford treatment, delivering orthodontic treatment after crowding has developed is not possible. However, preventive services are much cheaper and can be easily delivered by general dental practitioners ^6^. Although interceptive treatment techniques are simple in nature, a sound diagnosis is essential. Therefore, the ability to predict future crowding in a child is vital.

On the other hand, performing interceptive orthodontic procedures (such as serial extraction) at the right time is very important. The appropriate age for most interceptive interventions is when children are in the mixed dentition phase ^7^. To predict severe crowding in a child, clinicians often use some diagnostic clues such as the premature exfoliation of primary canines, prominent bulging in the canine area and the crescent area of root resorption in roots of primary canines ^8^. It is also proposed that the variations in teeth eruption may be an important aspect of crowding ^9^. Although a few studies have supposed averages or standards for tooth eruption sequence ^9, 10^, little has been done to understand the relationship between tooth eruption sequence variations and dental crowding.

Sampson and Richards, for instance, tested the hypothesis that pre-eruptive tooth positions might forecast crowding and proposed that a buccal eruption path of a mandibular canine indicates an insufficient space in the dental arch ^11^. Moorrees and Reed found that the utilization of leeway space depends on the sequence of shedding and eruption of the mandibular teeth ^12^. In another study, a low but significant correlation was found between increased crowding in the mandibular segment and the retardation of early phases of canine eruption ^13^. More recently, Lange has claimed that those children whose teeth eruption pattern does not follow the standard sequence are at greater risk of developing crowding ^14^. His study has been conducted on a confined population, therefore, it warrants further investigations to see whether the same results can be observed in other ethnics.

Considering the shortcomings of the literature in correlating the tooth eruption sequence to the possible lack of space, this case-control study was designed to better understand whether the mandibular teeth eruption sequence differs in patients with moderate to severe dental arch crowding.

## Materials and methods

A case-control study was designed and approved by the Shiraz University of Medical Sciences’ Orthodontic Research Center in Shiraz, Iran (Approval number 89-01-37-1940[8716]). Children aged from 8 to 10 years that were admitted to the Shiraz Dental School for orthodontic treatment during September to December of 2012 were enrolled in the study. Patients with a history of metabolic disease, nutrition deficiency, traumatic accidents to jaws and premature tooth loss as well as patients with missing data (broken plaster models, partially erupted anterior teeth and poor quality radiographs) were excluded from the study. The objectives and process of the study were explained to the parents. They were assured of the confidentiality of their and their children’s personal information. They were also assured that participation in this study (or their refusal) had no effect on their course of treatment. Parents were then asked to fill and sign the study’s informed consent form. Children of those who did not give their written consent were also removed from the study’s register. The final list of eligible patients consisted of 327 children. The next step was to divide the eligible children into two groups with and without crowding and to randomly select 36 children from each group.

**Figure 1.**
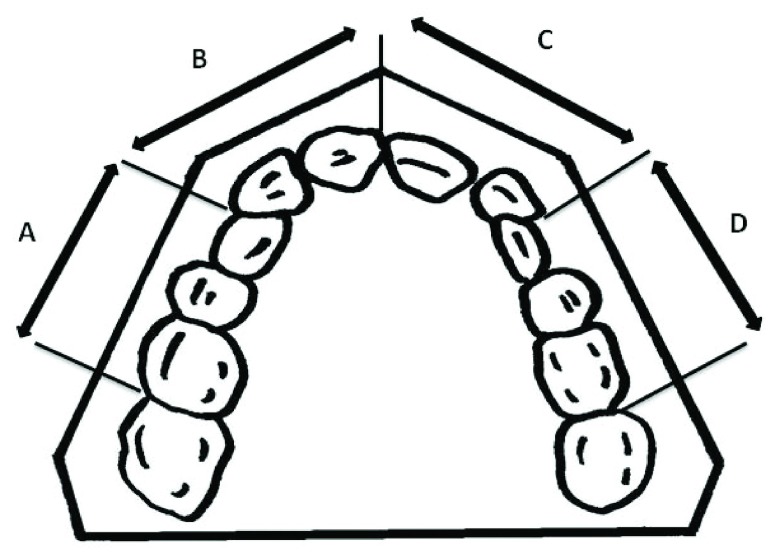
Dividing the dental arch into four segments for calculation of available space based on the method described by Sharma *et al.* ^15^.

Children’s plaster models, which were previously obtained for the purpose of orthodontic treatment, were assessed in a random order to calculate the tooth-size arch size discrepancy (TSALD). To do so, the space required for eruption of ten permanent anterior and premolar teeth was deducted from the space available from molar to molar teeth. The available space was measured by dividing the dental arch into four separate segments ( Figure 1) and summing them up, as previously described ^15^. The widths of mandibular incisors teeth, which were already erupted, were measured using a digital caliper with accuracy of 0.01 millimeter and were summed up. The width of un-erupted canine and premolar teeth was estimated using the Moyers table ^16^ and the Tanaka-Johnston formulae ^17^. The average of values gained from the two methods of estimation was used in the study.

The children were then divided into two groups based on their TSALD: those with a TSALD of equal or less than -4mm (crowding group), and those with a TSLAD of greater than -4mm (no crowding group). A computer generated random sample of 36 children was selected from the "crowding" group, as cases (TSLD≤-4mm). For each case, one subject matching sex and age was assigned as a control. The control assignment was conducted using a computer based randomization among the "no crowding" group (TSLD>-4mm). Therefore, data from a total of 72 children were used in the final analysis.

**Figure 2.**
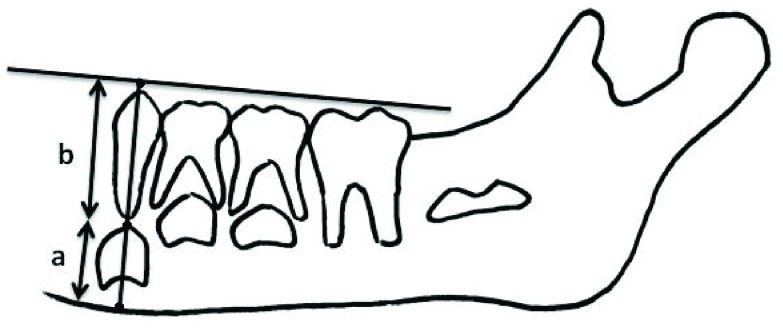
Calculating the eruption percentage for canines and first premolars $\left(percentage\;=\;\frac{b}{a\;+\;b}\;*\;100\right)$.

The final sample’s panoramic radiographies, which were previously taken for orthodontic treatment purposes, were traced to determine the eruption percent of mandibular canines and first premolar teeth. The tracing was made on acetate paper with a 3H pencil. As the eruption sequence is symmetrical in the left and right sides, tracing of only one side of the mandible was sufficient ^18^. A line was passed through the cusp tip of un-erupted permanent canine and the center of its predecessor primary canine ( Figure 2). The distance from the inferior border of the mandible to the cusp tip of un-erupted permanent canine was measured (a, Figure 2). Also, the distance from the cusp tip of un-erupted permanent canine to the line passing through the cusp tip of the primary molars and permanent first molar was measured (b, Figure 2). The eruption percent of canines was then calculated by dividing the first measurement by sum of both (a/a+b). A similar procedure was done to calculate the eruption percentage of permanent first premolar. The above-mentioned technique to calculate the eruption percentage of un-erupted teeth was adapted from Shumakher and El Hadary ^19^.

All measurements were performed twice by two experienced dentists (V.M. and N.K.). SPSS (version PASW 18) software was used for data entry and analysis. The Pearson correlation test was used to evaluate the reliability of measurements reported by the two assessors ( Table 1). The average of the two measurements was used in the final analysis. Paired sample t tests were used for statistical analyses.

**Table 1.** Pearson correlation coefficients for measurements obtained by the two assessors.

Measurement	R
**Total incisor width**	0.932
**Eruption percent on tracing**	0.903
**Available space (TSALD)**	0.934

## Results

Data from all 72 selected children were used in the final analysis. Sex and age distributions are shown in Table 2. The mean TSALD in the case and control groups was -6.22±2.49mm and 0.42±2.30mm, respectively. In the case group, canines, with the average eruption percentage of 65.82±13.00, were significantly behind the premolars which were on average 78.92±10.15 percent erupted (p<0.001). However, the average eruption percentage of canines (74.12±14.55) was very close to that of first premolars (75.47±11.60) in the control group (p=0.437) ( Table 3). Therefore, in the case group, which had greater TSALD than controls, the first premolar teeth would erupt before the canines, while there was no priority in the control group. The difference in eruption percent of canines and first premolar teeth between cases and controls was statistically significant (p<0.001).

**Table 2.** Number of samples in each age and sex group (36 cases and 36 controls).

	8-years-old		9-years-old		10-years-old		total	
	Case	Control	Case	Control	Case	Control	Case	Control
**Boys**	7	7	4	4	8	8	19	19
**Girls**	5	5	9	9	3	3	17	17
**Total**	12	12	13	13	11	11	36	36

**Table 3.** Comparison of canine and first premolar eruption percentages and their difference between case and control groups.

	Mean TSALD (mm)	Mean eruption % of canine	Mean eruption % of first premolar	Difference of canine and first premolar eruption (%)	Significance level of the difference between canine and first premolar eruption percentage
**Case**	-6.22±2.49	65.82±13.00	78.92±10.15	13.10±12.98	p<0.001
**Control**	0.42±2.30	74.12±14.55	75.47±11.60	1.35±10.29	p=0.437
**Significance level of** **the difference between** **case and control groups**	p<0.001	p=0.006	p=0.151	p<0.001

Tooth size-arch length discrepancies and teeth eruption in children with and without dental crowding The percentages of tooth size arch length discrepancies and teeth eruption in cases (TSALD<-4mm) and their matched controls (TSALD>-4mm) are reported in the data set. TSALD 1: tooth size-arch length discrepancy in cases; canine 1: eruption percentage of permanent canines in cases; premolar 1: eruption percentage of first premolar in cases; TSALD 2: tooth size-arch length discrepancy in matched controls; canine 2: eruption percentage of permanent canines in matched controls; premolar 2: eruption percentage of first premolar in matched controls.

## Discussion

This study was designed and conducted based on the hypothesis that the eruption sequence of the permanent canines and premolars of children with dental crowding differs from that of children without dental crowding. To the author’s knowledge, this is one of the first studies reporting a clear correlation between canine and premolar eruptive position in the mixed dentition stage and dental crowding. The results showed that there was a significant difference in the pre-eruptive positions of canine and first premolar teeth in cases with moderate to severe crowding compared to controls. The difference between the groups was large enough to be clinically detected. Clear clinical differences between canine and first premolar eruption order could be detected by a cursory assessment of panoramic radiographies and seemed to confirm the results. Therefore, canine and premolar eruption order assessed on radiographies can be used to identify children with high risk of developing moderate to severe crowding in mixed dentition.

In our study, we selected children from 8 to 10 years old age and compared the eruption percentage of two teeth, namely canine and first premolar in the mandible. The age group selected is corresponding to the mixed dentition age when most of orthodontic interceptions could be effective. Often crowding is more severe in the mandibular arch as there are several mechanisms to resolve the lack of space in the maxilla ^11– 14^.

We observed that patients with dental crowding presented a possible delay in the eruption of their permanent mandibular canines compared to their adjacent premolars. This observation was compatible with Bradley’s notation of retardation in the eruption time of mandibular canines when there was a lack of space ^13^. Lange has also found that more crowding is observed with the eruption sequence of 4-3-5 compared with 3-4-5 (i.e. when the first premolar erupts before canine), a finding that is exactly the same as ours ^14^.

It has been shown in the literature that the utilization of leeway space is indicative of crowding ^20, 21^. Moorrees also concludes that utilization of leeway space depends on the sequence of eruption and shedding of posterior teeth. We can therefore justify our findings on the basis that eruption of the first premolar before the canine teeth may result in using the leeway space inappropriately which increases the chance of development of dental crowding ^12^.

Our study suggests a practical approach for early identification of children susceptible to develop dental crowding. Just two teeth were assessed. That made this study different from other similar studies in which too many variables from several teeth were considered ^11, 13^. As a result, a simple significant difference was found in the present study that can act as a practical clue for clinicians.

We used TSALD measurements in the total arch as the indicator of crowding. This is another advantage of this study over the few similar ones that only calculated the crowding in the canine-premolar segment ^13^. Most crowding in the mandible occurs in the incisor-canine segment and in central-lateral contact first and then migrates to posterior segments ^22, 23^.

Clinicians are often confronted with decisions concerning the choice of interceptive treatments for potential crowding during mixed dentitions. Extreme caution should be exercised in selecting patients that will truly benefit from interceptive procedures such as planned extractions. The findings of the present study can be used together with other clues to select suitable cases for such treatments.

## Conclusion

The findings of this study showed that children whose first premolar teeth precede their canines in eruption are more likely to develop malocclusions related to TSALD later on. Therefore, routine screening of the panoramic views of children seeking orthodontic consultation in mixed dentition would be helpful in the diagnosis of children with the chance of developing moderate to severe crowding in their permanent dentition.

## Data availability

F1000Research: Dataset 1. Tooth size-arch length discrepancies and teeth eruption in children with and without dental crowding, 10.5256/f1000research.3196.d27729 ^24^

## Consent

Written informed consent for publication of clinical details was obtained from the parents of the children.
