# Supplementary material for: Tooth eruption sequence and dental crowding: a case-control study
Source: F1000Res. 2014 Jun 6;3:122. [Version 1] doi: 10.12688/f1000research.3196.1 (PMC4111114; doi:10.12688/f1000research.3196.1)
Supplement: Peer review history

# Peer review: Referee response for version 1

**DOI:** 10.5256/f1000research.3436.r5035

**Published:** 2014-06-12

**Authors:** Mahtab Nouri

**Affiliations:** 1 School of Dentistry, Shaheed Beheshti University, Tehran, Iran

This is an interesting paper about the role of  sequence and percentage of eruption of first premolar in comparison to canine on development of crowding in the mandible. This case control study has a sound research methodology. I wished that there was an explanation added about the dataset regarding the control group and the method of its TSALD calculation. The conclusion is justified.

I have read this submission. I believe that I have an appropriate level of expertise to confirm that it is of an acceptable scientific standard.

# Peer review: Referee response for version 1

**DOI:** 10.5256/f1000research.3436.r5046

**Published:** 2014-06-09

**Authors:** Wael Sabbah

**Affiliations:** 1 Department of Dental Public Health, King's College London, London, UK

This is a very good paper, methodologically sound. The findings of the paper have the potential to allow early detection of malocclusion cases and would possibly benefit treatment planning for orthodontic cases.

A minor change is needed. In the first paragraph the authors referred to 'NHANES III 1998'. The date for NHANES III is 1988-1994. If the authors are referring to another year or to NHANES in general they should amend the text and use the reference.

I have read this submission. I believe that I have an appropriate level of expertise to confirm that it is of an acceptable scientific standard.
